# The safety and efficacy of Ringer's solution loading with rectal diclofenac for prevention of post‐endoscopic retrograde cholangiopancreatography pancreatitis: The RESOLUTION‐PEP study

**DOI:** 10.1002/deo2.236

**Published:** 2023-04-26

**Authors:** Aiji Hattori, Reiko Yamada, Toji Murabayashi, Shinya Sugimoto, Hajime Imai, Keiichiro Nojiri, Naoki Kuroda, Shunsuke Tano, Hiroki Tanaka, Shimpei Matsusaki, Kenichiro Nishikawa, Hiroaki Naota, Isao Moritani, Kazunari Kurata, Toshitaka Fukui, Kyosuke Tanaka, Hayato Nakagawa

**Affiliations:** ^1^ Department of Gastroenterology and Hepatology Mie University Hospital Mie Japan; ^2^ Department of Gastroenterology Matsusaka Municipal Hospital Mie Japan; ^3^ Department of Gastroenterology Ise Red Cross Hospital Mie Japan; ^4^ Department of Gastroenterology Okanami General Hospital Mie Japan; ^5^ Department of Gastroenterology Kuwana City Medical Center Mie Japan; ^6^ Department of Gastroenterology Saiseikai Matsusaka General Hospital Mie Japan; ^7^ Department of Gastroenterology Suzuka Kaisei Hospital Mie Japan; ^8^ Department of Gastroenterology Suzuka General Hospital Mie Japan; ^9^ Department of Gastroenterology Matsusaka Chuo General Hospital Mie Japan; ^10^ Department of Gastroenterology Mie Prefectural General Medical Center Mie Japan; ^11^ Department of Gastroenterology Mie Chuo Medical Center Mie Japan; ^12^ Department of Gastroenterology Yokkaichi Hazu Medical Center Mie Japan; ^13^ Department of Endoscopic Medicine Mie University Hospital Mie Japan

**Keywords:** anti‐inflammatory agents, endoscopic retrograde cholangiopancreatography, non‐steroidal, pancreatitis, prevention

## Abstract

**Objectives:**

We evaluated the safety and efficacy of aggressive hydration with rectal non‐steroidal anti‐inflammatory drugs for the prevention of post‐endoscopic retrograde cholangiopancreatography (ERCP) pancreatitis (PEP).

**Methods:**

This prospective, single‐arm, multicenter trial was conducted at 12 institutions between October 2020 and August 2021. We enrolled 231 patients who had intact papillae and were scheduled to undergo ERCP. All patients were administered rectal diclofenac before ERCP. They received aggressive hydration with intravenous lactated Ringer's solution in an initial bolus of 5 ml/kg at the start of ERCP, followed by 3 ml/kg/h for 8 h after the procedure. The primary outcome was the occurrence of PEP. Secondary outcomes included PEP severity, hyperamylasemia, and adverse events.

**Results:**

The mean age of the patients was 68.8 ± 13.7 years, and 81 patients (35.1%) were 75 years or older. Thirteen patients developed PEP (5.6%, 95% confidence interval 3.0%–9.4%). There were 11 cases (4.8%) of mild pancreatitis and two cases (0.9%) of severe pancreatitis. Forty‐five patients (19.5%) developed hyperamylasemia and one patient developed non‐severe peripheral edema.

**Conclusions:**

Aggressive hydration combined with rectal diclofenac may be a promising strategy for the prevention of PEP. Furthermore, it is safe even for older individuals.

## INTRODUCTION

Endoscopic retrograde cholangiopancreatography (ERCP) is an important procedure to diagnose and treat diseases of the pancreaticobiliary system. Post‐ERCP pancreatitis (PEP) is the most common adverse event (AE) of ERCP. In a systematic review of patients enrolled in a randomized controlled trial (RCT), the incidence of PEP was 9.7% in all patients, and 14.7% in high‐risk patients.^1^ To reduce the risk of PEP, numerous prophylactic measures have been studied for the prevention of PEP. Previous studies reported a prophylactic pancreatic stent could reduce the risk of PEP in high‐risk patients.^2^, ^3^, ^4^

Pharmacological studies have been reported for the prevention of PEP. Non‐steroidal anti‐inflammatory drugs (NSAIDs) are potent inhibitors of phospholipase A2, cyclooxygenase, and neutrophil–endothelial interactions, which were suggested as having roles in the pathogenesis of acute pancreatitis.^5^, ^6^ Several studies conducted in Western countries showed a 50–100‐mg rectal dose of NSAIDs could prevent PEP.^7^, ^8^ However, a 100‐mg dose of NSAIDs is higher than the usual dose (25–50 mg) in Japan, and the severity of certain complications increases with a higher NSAID dose.^9^ Other reports showed 25–50 mg rectal diclofenac could also prevent PEP.^10^, ^11^

The use of an infusion of lactated Ringer's solution (LRS) appears to have an important role in the prevention of pancreatic necrosis and organ failure by maintaining stable pancreatic microcirculation.^12^, ^13^, ^14^, ^15^ Aggressive hydration with LRS has shown a benefit in the prevention of PEP.^16^, ^17^

For the prevention of PEP, few studies have evaluated the possible benefits of aggressive hydration with a rectal dose of NSAIDs.^18^, ^19^ In this multicenter study, including elderly patients, we conducted a prospective single‐arm trial to evaluate the safety and efficacy of aggressive hydration with rectal NSAIDs for the prevention of PEP.

## METHODS

### Study design

A prospective multicenter, single‐arm trial was performed in average‐ to high‐risk patients who underwent ERCP between October 2020 and August 2021 at 12 institutions in Mie prefecture, Japan. All the respective institutional review boards approved the study protocol, and the study was conducted in accordance with the Declaration of Helsinki (permission number: H2020‐138). Written informed consent was obtained from all patients before commencing study participation and undergoing ERCP.

### Patients

Eligible patients who had intact papillae and were scheduled to undergo ERCP were included in this study. The exclusion criteria included: (1) age younger than 20 years; (2) heart failure higher than New York Heart Association class II; (3) renal insufficiency (eGFR <45 ml/min/1.73 m^2^); (4) respiratory failure (SpO_2_ < 90% in room air); (5) concomitant acute pancreatitis before ERCP; (6) performance status above a score of 3; (7) presence of a stoma that ruled out rectal NSAIDs; (8) other contraindications for administering NSAIDs; (9) a history of gastrectomy with Billroth I/II, or Roux‐en‐Y reconstruction; (10) inability to reach the papilla because of gastroduodenal obstruction; (11) pregnancy or the possibility of pregnancy; and (12) patients considered inappropriate for the trial. After informed consent was obtained, all patients were administered rectal diclofenac before ERCP. The dose of diclofenac was 50 mg basically, but it was accepted to administer a 25‐mg diclofenac if the patient whose body weight was under 50 kg.

### Procedure

ERCP was performed using a side‐viewing endoscope (JF‐260V and TJF‐260V, TJF‐Q290V; Olympus Medical Systems, Tokyo, Japan) with standard cannulation techniques. During ERCP, patients under continuous sedation were continuously monitored for blood pressure, heart rate, and oxygen saturation. The definition of the procedure time was from insertion to removal of the endoscope. The patient received aggressive hydration with intravenous LRS in an initial bolus of 5 ml/kg concurrently at the start of ERCP and continuously during the procedure. Then, hydration with intravenous LRS continued at a rate of 3 ml/kg/h for 8 h after the procedure. To monitor for AEs, routine blood examinations with complete blood count, and biochemistry including serum amylase levels and C‐reactive protein were performed twice when before the procedure and 8–18 h after the procedure. In patients with suspected/definite PEP, computed tomography was performed as needed, and standard PEP treatment (e.g., infusion of a large volume of fluid, protease inhibitors, and antibiotics) was administered.

### Study outcome and definition

The primary outcome was the incidence of PEP. The diagnosis of PEP was defined as new or worsening epigastric pain lasting at least 24 h after ERCP and an increase in pancreatic enzymes greater than three times the upper limit of normal within 24 h after ERCP.^1^, ^20^

Secondary outcomes were the severity of PEP, the occurrence of hyperamylasemia, and the AEs of aggressive hydration with diclofenac. The severity of PEP was defined according to the revised Atlanta classification. Mild acute pancreatitis was defined as no organ failure or local or systemic complications. Moderate acute pancreatitis was defined as organ failure that resolved within 48 h and/or local or systemic complications. Severe acute pancreatitis was defined as a persistent organ failure not resolved within 48 h.^21^ The occurrence of hyperamylasemia was defined as serum amylase levels of at least three times the upper normal limit within 24 h without epigastric pain. All patients were monitored after the procedure and underwent clinical assessment for signs of fluid overload (e.g., tachypnea, abnormal breath, distension of the jugular veins, and lower extremity edema). To evaluate the safety of this combination therapy, we assessed AEs such as gastrointestinal bleeding, peripheral/pulmonary/cerebral edema, and congestive heart failure. Other post‐ERCP complications such as hypoxia, hypotension, bleeding, perforation, and cholangitis were also recorded.^22^ High‐risk patients in this study were defined as having one or more of the risk factors of PEP. The risk factors were as follows: (1) female sex, (2) younger patient age (<50 years), (3) suspected sphincter of Oddi dysfunction (SOD), (4) difficult cannulation (>10 min), (5) pancreatic duct injection, (6) pancreatic sphincterotomy, (7) precut sphincterotomy, and (8) biliary sphincter balloon dilation. Difficult cannulation was defined as prolonged duration before cannulation (>10 min).^1^ SOD is defined as symptoms of pancreaticobiliary obstruction in the absence of other structural causes such as malignancy or stones.

### Sample size calculation

On the basis of the incidence rates of PEP in the previous report, we assumed the incidence of pancreatitis would be 11.1% in the standard group and would decrease to 5.1% in the active hydration group.^23^ Therefore, it's estimated approximately 180 patients would be needed, with an 11% incidence of PEP, an expected value of 5%, and alpha (one‐sided) and beta values of 0.025 and 0.2, respectively. The consent rate for participation in the study was estimated to be 80%, which would ultimately require approximately 230 patients.

### Statistical analysis

We evaluated the characteristics and AEs of the elder and non‐elder patient groups. In addition, the baseline patient characteristics and risk factors were compared between the PEP and non‐PEP groups. Continuous variables were presented as means with standard deviations or medians with interquartile ranges using Student's *t*‐test. Fisher's exact test was used for the statistical analysis of categorical variables. The logistic regression and stepwise selection model were used to calculate the adjusted odds ratio with a 95% confidence interval (CI) for the risk of PEP. The multivariate logistic regression model was performed with adjustments for all patient‐and‐procedure‐related risk factors as listed in Table 1. The threshold for significance was *p* < 0.05. All statistical analyses were conducted using EZR,^24^ which is a graphical user interface for R. More precisely, it's a modified version of R commander designed to add statistical functions frequently used in biostatistics.

**TABLE 1:** Baseline characteristics of all patients.

**Number of patients (*n*)**	**231**	
Age (mean ± SD)	68.8	±13.7
Body mass index (mean ± SD)	23.2	± 4.1
Older patients*1 (*n*)	81	(35.1%)
Use of antithrombotic agent (*n*)	37	(16.0%)
Administration of 50‐mg diclofenac (*n*)	206	(89.2%)
Cholangitis before ERCP (*n*)	42	(18.2%)
High‐risk patient*2 (*n*)	185	(80.1%)
Indications (*n*)		
.Choledocholithiasis	116	(50.2%)
.Malignancy	83	(35.9%)
.Others	32	(13.9%)
Endoscopic procedure		
.Cannulation time (min), median (IQR)	9	(3–18)
.Number of cannulations, median (IQR)	5	(2–15)
.EST (*n*)	170	(73.6%)
.Balloon dilation (EPBD/EPLBD) (*n*)	25	(10.8%)
.Biliary stent placement (*n*)	109	(47.2%)
.Pancreatic stent placement (*n*)	37	(16.0%)
Patient‐related risk factors (*n*)		
.Suspected SOD	9	(3.9%)
.Female sex	98	(42.4%)
.Younger patient age (<50 years old)	23	(10.0%)
Procedure‐related risk factors (*n*)		
.Difficult cannulation*3	96	(41.6%)
.Pancreatic sphincterotomy	4	(1.7%)
.Precut sphincterotomy	18	(7.8%)
.Balloon dilation (EPBD/EPLBD)	25	(10.8%)
.Pancreatic duct injection	110	(47.6%)

Abbreviations: EPBD, endoscopic papillary balloon dilation; EPLBD, endoscopic papillary large balloon dilation; EST, endoscopic sphincterotomy; IQR, interquartile range; SD, standard deviation; SOD, sphincter of Oddi dysfunction.

*1 Patients aged 75 years or older.

*2 Defined as high‐risk patients who had one or more of the risk factors.

*3 Defined as prolonged duration before cannulation (>10 min).

## RESULTS

### Baseline characteristics

During the study period, 241 patients were assessed for eligibility. Ten of the patients were excluded because of infusion load errors (*n* = 7) or duodenal obstruction recognized during ERCP (*n* = 3). Ultimately, 231 patients were enrolled in the study. The baseline characteristics of the patients are shown in Table 1. The mean age of the patients was 68.8 years (standard deviation, 13.7 years), and 98 patients (42.4%) were female. Eighty‐one patients (35.1%) were aged 75 years or older. The major indications for ERCP were choledocholithiasis (57.3%) and malignancy (35.9%).

Endoscopic sphincterotomy was performed in 170 patients (73.6%), balloon dilation in 25 patients (10.8%), and biliary stent placement (plastic or metallic stent) in 109 patients (47.2%). The major risk factors for PEP are shown in Table 1. One hundred eighty‐five patients (80.1%) had one or more of the risk factors and were defined as high‐risk patients.

### Study outcomes

The primary outcome of PEP occurred in 13 of 231 patients (5.6%, 95% CI 3.0%–9.4%). Their mean age was 65.0 ± 17.2 years, and 12 cases in the PEP group (12/13, 92.3%) were high‐risk patients. Eleven patients (4.8%) had mild PEP. Two patients (0.9%) had severe PEP. Hyperamylasemia was observed in 45 patients (19.5%), and abdominal pain in 24 patients (10.4%). In AEs related to the ERCP procedure, there were three patients with hypoxia (1.3%), six patients with hypotension (2.6%), one patient with bleeding (0.9%), two patients with perforation (0.9%), and five patients of cholangitis (2.2%). There were two fatalities (0.9%) due to ERCP procedures: one due to severe PEP and the other due to PEP and perforation. In AEs related to hydration with diclofenac, there was one case (0.9%) of peripheral edema, but this wasn't severe. There're no instances of gastrointestinal bleeding, pulmonary or cerebral edema, or congestive heart failure (Table 2).

**TABLE 2:** Adverse events of all patients.

**AEs related to ERCP procedure (*n*)**		
PEP	13	(5.6%)
.Mild	11	(4.8%)
.Moderate	0	(0.0%)
.Severe	2	(0.9%)
Hyperamylasemia	45	(19.5%)
Abdominal pain	24	(10.4%)
Hypoxia*1	3	(1.3%)
Hypotension*2	6	(2.6%)
Bleeding	1	(0.4%)
Perforation	2	(0.9%)
Cholangitis	5	(2.2%)
Deaths due to ERCP procedure	2	(0.9%)
PEP	1	(0.4%)
PEP and perforation	1	(0.4%)
AEs related to hydration with diclofenac (*n*)		
Gastrointestinal bleeding	0	(0.0%)
Pulmonary edema	0	(0.0%)
Cerebral edema	0	(0.0%)
Peripheral edema	1	(0.4%)
Congestive heart failure	0	(0.0%)
Death from hydration with diclofenac	0	(0.0%)

Abbreviations: AEs, adverse events; ERCP, endoscopic retrograde cholangiopancreatography; PEP, post‐endoscopic retrograde cholangiopancreatography pancreatitis.

*1 Oxygen saturation <90%.

*2 Blood pressure <90/50 mmHg.

### Subgroup analysis

Table 3 compares the characteristics and AEs between the older and non‐older patient groups in a univariate analysis. The usage rate of antithrombotic agents in older patients was higher (*p* < 0.05) and the frequency of 50‐mg diclofenac administration was lower (*p* < 0.001) than that in non‐older patients. There're no differences between the older and non‐older groups regarding indications or the number of high‐risk patients. There're also no differences between the two groups regarding either AEs related to the ERCP procedure or hydration with diclofenac.

**TABLE 3:** Comparison of baseline characteristics and adverse events (AEs) of older patients and non‐older patients by univariate analysis.

	**Elder patients (*n*=81)**		**Non‐elder patients (*n*=150)**		***p*‐Value**
Baseline characteristics			
Age (mean ± SD)	81.7 ± 4.5		61.9 ± 11.8		<0.00001*
Body mass index (mean ± SD)	22.4 ± 3.2		23.5 ± 4.4		0.037*
Female sex (*n*)	36	(44.4%)	62	(41.3%)	0.677^†^
Use of antithrombotic agent (*n*)	25	(30.9%)	12	(8.0%)	0.00001^†^
Administration of 50‐mg diclofenac (*n*)	67	(82.7%)	139	(92.7%)	0.026^†^
Choledocholithiasis (*n*)	38	(46.9%)	78	(52.0%)	0.492^†^
Malignancy (*n*)	36	(44.4%)	47	(31.3%)	0.061^†^
Suspected SOD (*n*)	3	(3.7%)	6	(4.0%)	1.000^†^
AEs (*n*)					
PEP	5	(6.2%)	8	(5.3%)	0.773^†^
Hyperamylasemia	12	(14.8%)	33	(22.0%)	0.226^†^
Abdominal pain	8	(9.9%)	16	(10.7%)	1.000^†^
Hypoxia	1	(1.2%)	2	(1.3%)	1.000^†^
Hypotension	1	(1.2%)	5	(3.3%)	0.668^†^
Bleeding	0	(0.0%)	1	(0.6%)	1.000^†^
Perforation	1	(1.2%)	1	(0.6%)	1.000^†^
Cholangitis	1	(1.2%)	4	(2.7%)	0.660^†^
Peripheral edema	0	(0.0%)	1	(0.6%)	1.000^†^
Deaths from PEP	1	(1.2%)	1	(0.6%)	1.000^†^

Abbreviations: AEs, adverse events; SD, standard deviation; SOD, sphincter of Oddi dysfunction.

* Student's t‐test.

^†^ Fisher's exact test.

Table 4 shows the baseline patient characteristics of the PEP and non‐PEP groups. The mean age of the PEP group was 65.0 years (standard deviation, 17.2 years), and 46.2% were female. The frequency of SOD, difficult cannulation, and pancreatic duct injection was higher in the PEP group than in the non‐PEP group (*p* < 0.05).

**TABLE 4:** Comparison of baseline characteristics and risk factors between the post‐endoscopic retrograde cholangiopancreatography pancreatitis (PEP) and non‐PEP groups by univariate and multivariate analysis.

	**PEP group (*n* = 13)**		**Non‐PEP group (*n* = 218)**		**Univariate analysis OR (95% CI) *p*‐value**		**Multivariate analysis** * **OR (95% CI) *p*‐value**	
Risk factors (*n*)						
Female sex	6	(46.2%)	92	(42.2%)	1.2 (0.4–3.6)	0.78		
Suspected SOD	3	(23.1%)	6	(2.8%)	10.6 (2.3–48.7)	0.0024	7.2 (1.5–35.0)	0.014
Younger patient (<50 years old)	3	(23.1%)	20	(9.2%)	3.0 (0.8–11.7)	0.12		
Difficult cannulation	10	(76.9%)	86	(39.4%)	5.1 (1.4–19.1)	0.015	4.2 (1.1–16.3)	0.036
Balloon dilation (EPBD/EPLBD)	2	(15.4%)	23	(10.6%)	1.5 (0.3–7.4)	0.59		
Pancreatic sphincterotomy	1	(7.7%)	3	(1.4%)	6.0 (0.58–61.8)	0.13		
Precut sphincterotomy	1	(7.7%)	17	(7.8%)	1.0 (0.1–8.0)	0.99		
Pancreatic injection	10	(76.9%)	100	(45.9%)	3.9 (1.1–14.7)	0.042		

Abbreviations: AEs, adverse events; CI, confidence interval; EPBD, endoscopic papillary balloon dilation; EPLBD, endoscopic papillary large balloon dilation; OR, odds ratio; PEP, post‐endoscopic retrograde cholangiopancreatography pancreatitis; SOD, sphincter of Oddi dysfunction.

* Logistic regression analysis (stepwise regression).

## DISCUSSION

In this study, the rate of PEP was 5.6% in patients who received aggressive hydration combined with low‐dose (25–50 mg) diclofenac. To our knowledge, there're no reports that evaluate the efficacy of aggressive hydration with low‐dose rectal diclofenac for the prevention of PEP. Furthermore, despite the inclusion of older patients, there're almost no AEs resulting from volume overload with aggressive hydration. Aggressive hydration combined with low‐dose diclofenac may be a promising and safe strategy for the prevention of PEP.

Previous studies have revealed the effectiveness of rectal administration of NSAIDs in preventing PEP, and The European Society of Gastrointestinal Endoscopy (ESGE) recommends routine rectal administration of 100‐mg diclofenac or indomethacin before ERCP.^25^ However, in Japan, the usual dose of NSAIDs is 25–50 mg, and rectal administration of 100‐mg diclofenac or indomethacin isn't recommended. Otsuka et al. revealed the efficacy of low‐dose diclofenac in preventing PEP in an RCT. They reported 12/104 (11.5%) patients developed PEP, and the incidence of PEP was lower in patients who received 50‐mg rectal diclofenac compared with the control group (2/51 [3.9%] vs. 10/53 [18.9%]; *p* = 0.017).^10^ Okuno et al. reported the incidence of PEP in patients treated with 25‐mg rectal diclofenac was lower than that in the control group (3/74 [4.1%] vs. 10/73 [13.7%]; *p* = 0.046).^11^ Both studies reported no AEs related to diclofenac.

Another preventive strategy is aggressive hydration. In the early phase of PEP development, there's a microcirculatory impairment due to increased parenchymal pressure. Intravenous fluid substitution maintains perfusion of the small vessels, repairs impaired fluid distribution, and reduces the risk of hypoxia, reactive oxygen species generation, and mitochondrial injury.^26^ Previous studies have suggested the possibility of aggressive hydration to prevent PEP by improving pancreatic microvasculature viability and preventing pancreatic acidification, zymogen activation, and initiation of the inflammatory cascade.^12^, ^13^, ^14^, ^15^ Several meta‐analyses of RCTs reported aggressive hydration appeared to be an effective and safe strategy for the prevention of PEP.^16^, ^17^, ^27^, ^28^

Several studies reported the benefits of this combination therapy in RCTs. Hosseini et al. compared the following four groups to examine combined fluid and NSAID treatment: (1) rectal indomethacin; (2) intravenous (IV) saline perfusion; (3) combined rectal indomethacin and IV saline: and (4) placebo. They revealed that hydration combined with NSAID treatment significantly lowered the incidence of PEP compared with the placebo (0/101 [0%] vs. 17/105 [16.2%]; *p* < 0.001).^18^ Mok et al. evaluated the efficacy of indomethacin with or without bolus LRS in high‐risk patients for PEP. The incidence of PEP in the LRS group was lower than that in the placebo group (3/48 [6%] vs. 10/48 [21%]; *p* = 0.04).^19^ Conversely, Weiland et al. compared the combination of aggressive hydration and rectal NSAIDs (100 mg) with rectal NSAIDs alone (control group) and revealed that the combined treatment didn't reduce the incidence of PEP (30/388 [8%] vs. 39/425 [9%]; *p* = 0.53).^29^ However, in their report, the control group received intravenous saline at a maximum of 1.5 ml/kg/h and 3 L per 24 h, which might be considered sufficient hydration. This may explain the lack of difference between the combination therapy group and the control group.

In the present study, the PEP rate was 5.6% (13/231, 95% CI 3.0%–9.4%), which was similar to that reported in previous studies. In high‐risk patients in particular (80.1%), the PEP rate was 6.5% (12/185, 95% CI 3.4%–11.1%), which was significantly lower than that in a previous study (14.7%).^1^ This may be indicative of the potential beneficial effect of using a combination of aggressive hydration with NSAID therapy for high‐risk patients. Furthermore, in this study, older patients were included without an upper age limit. The severity of NSAID‐related complications is dependent on the duration and dose of NSAID use, and the administration of NSAIDs to older people increases the risk of complications.^9^ Older patients may be more susceptible to overhydration because of comorbidities and age‐related declines in organ function.^30^ The ESGE cautioned against using aggressive hydration for older patients because of the higher risk of cardiovascular comorbidities.^25^ Our study included 81 patients (35.1%) aged 75 years or older. None of them had AEs related to diclofenac. There was one patient (0.9%) with peripheral edema due to overhydration, but the patient wasn't older, and the edema wasn't severe. Moreover, there were no significant differences between the older and non‐older patient groups with regard to other ERCP procedure complications. These results indicate aggressive hydration with low‐dose rectal NSAIDs is safe in older patients without major comorbidities. In this study, there were two deaths (0.9%) due to ERCP procedures. These two patients had multiple high‐risk factors (SOD, cannulation difficulty, pancreatic duct injection, and biliary sphincter balloon dilation). One patient had a perforation in addition to PEP and was over 80 years old, which may have reduced his reserve for AEs. In this case, it was unclear whether the cause of death was due to perforation or PEP. The other death was due to severe PEP. This patient was overweight (body mass index: 36.0%), which may have influenced prognostic factors for severe PEP. Previous studies have reported mortality rates of 0.1%–0.7% for PEP,^25^, ^31^ and the PEP‐only mortality rate in our study (0.4%) was similar to previous reports.

Previous studies reported the risk factors for PEP were both patient‐related factors and procedure‐related factors. All patient‐ and procedure‐related risk factors in this study were independent risk factors for PEP.^8^, ^22^, ^32^ In our subgroup analysis, SOD and difficult cannulation were likely to be PEP risks.

There are several limitations to this study. First, patients were enrolled according to strict inclusion criteria to ensure their safety for receiving aggressive hydration with rectal NSAIDs. The use of these strict inclusion criteria excluded patients with major comorbidities. These criteria may cause selection bias and limit the generalization of these results. Whereas, patients considered inappropriate for the trial were excluded at the discretion of the researcher in charge. This might have caused excluding the appropriate patients such as healthy elder patients. Second, because of the single‐arm design, we couldn't perform a statistical analysis of the effectiveness and safety of aggressive hydration with rectal NSAIDs. RCT is required to determine the effectiveness and safety of this combination therapy. Third, as a result of the small sample size of the PEP group, we couldn't undertake multivariate analyses of independent risk factors for PEP to demonstrate statistical associations. We evaluated the baseline patient characteristics and risk factors of PEP between the PEP and non‐PEP groups in the univariate analysis, but the results may include some confounding data.

In conclusion, aggressive hydration combined with low‐dose diclofenac may be a rational and promising strategy for the prevention of PEP. Furthermore, it's thought to be safe even for older individuals. RCT is required to validate the outcomes of our preliminary study.

## CONFLICT OF INTEREST STATEMENT

None.

## PATIENT CONSENT

Informed consent was obtained from the patients for the publication of their information and imaging.
